# Neuropilin-1 enriched mesenchymal stem cell derived-exosomes alleviate vascular hyperpermeability in acute lung injury

**DOI:** 10.20517/evcna.2025.169

**Published:** 2026-06-16

**Authors:** Qinyi Deng, Yifan Lu, Linyan Yuan, Shiyu Gao, Xiaoyan Hu, Van Minh Le, Xiaoyan Chen, Xin Liang, Yun Feng

**Affiliations:** ^1^Department of Respiratory and Critical Care Medicine, Ruijin Hospital, School of Medicine, Shanghai Jiao Tong University, Shanghai 200025, China.; ^2^State Key Laboratory of Bioreactor Engineering & Shanghai Key Laboratory of New Drug Design, School of Pharmacy, East China University of Science and Technology, Shanghai 200237, China.; ^3^Research Center for Discovery and Development of Healthcare Products, Vietnam National University Ho Chi Minh City, Ho Chi Minh City 700000, Vietnam.; ^4^Department of Pathology, Ruijin Hospital, School of Medicine, Shanghai Jiao Tong University, Shanghai 20025, China.; ^#^These authors contributed equally to this work.

**Keywords:** Mesenchymal stem cells, exosome, neuropilin1, ALI, vascular permeability

## Abstract

**Aim:** Mesenchymal stem cells (MSCs) have shown therapeutic potential in acute lung injury (ALI); however, the key functional components and their roles in regulating pulmonary vascular endothelial permeability remain unclear. This study aimed to identify functional proteins within MSC-derived exosomes (MSC-Exo) and elucidate their roles in regulating pulmonary vascular endothelial permeability to enhance MSC-Exo-based therapy.

**Methods:** Proteomic analysis identified neuropilin-1 (NRP1) as a candidate functional protein in MSC-Exo. Functional assays, including annexin V flow cytometry for apoptosis, colony formation assays for proliferation, and Transwell migration and wound healing assays, were performed to assess these processes in injured pulmonary microvascular endothelial cells (PMVECs). Immunofluorescence and Western blotting were used to evaluate NRP1 localization, p130Cas phosphorylation and matrix metalloproteinases 1 (MMP1)/9 expression. In ALI rat models, histopathology, Evans blue extravasation, and immunohistochemistry were used to assess lung injury and vascular permeability.

**Results:** NRP1-enriched MSC-Exo reduced apoptosis, enhanced colony formation, and promoted migration in PMVECs. Mechanistically, NRP1 modulated the Bcl-2/Bax ratio, inhibited caspase 3/9 activation, and promoted p130Cas phosphorylation with increased MMP1/9 expression. NRP1 also colocalized with PDGFR-α, suggesting a potential role in PDGF-BB signaling. *In vivo*, NRP1-overexpressing exosome showed superior efficacy in reducing pulmonary edema, vascular leakage, and restoring tight junction proteins, whereas NRP1 depletion impaired these effects.

**Conclusion:** NRP1 in MSC-Exo enhances endothelial repair by promoting proliferation and migration while inhibiting apoptosis. *In vivo*, NRP1-enriched MSC-Exo improve vascular barrier function and attenuate lung injury, supporting their therapeutic potential in ALI.

## INTRODUCTION

Acute lung injury (ALI), a localized pulmonary manifestation of systemic inflammation, is characterized by sudden hypoxemia, diffuse infiltrates, and increased vascular permeability leading to pulmonary edema. Its severe form, acute respiratory distress syndrome (ARDS), is a major cause of acute respiratory failure^[1]^. The core pathology involves disruption of the alveolar-capillary barrier driven by excessive inflammation, resulting in interstitial injury, protein-rich edema, and impaired gas exchange^[2]^. Despite advances in lung-protective ventilation strategies, abnormal alveolar dynamics may increase the risk of ventilator-induced lung injury (VILI), and mortality remains high due to the lack of effective pharmacological therapies^[3,4]^. At the molecular and cellular levels, this barrier dysfunction is largely attributed to endothelial injury. Inflammatory mediators and endotoxins trigger endothelial activation, leading to cytoskeletal rearrangement, increased vascular permeability, and disruption of intercellular junctions. In addition, oxidative stress and endothelial apoptosis further exacerbate barrier breakdown and vascular leakage, ultimately contributing to pulmonary edema and impaired gas exchange.

Tight junction (TJ) proteins - particularly zonula occludens-1 (ZO-1) and occludin (OCLN) - are vital to the structural integrity of pulmonary endothelial barriers. In ALI, pro-inflammatory cytokines and endotoxins such as lipopolysaccharide (LPS) disrupt the localization and expression of these junctional proteins, leading to inter-endothelial gap formation and increased vascular leakage. Restoration of TJ proteins has been shown to mitigate endothelial hyperpermeability and lung edema. Notably, MSC-derived exosomes (MSC-Exo) can enhance ZO-1 and OCLN expression via microRNAs (miRNAs) or protein cargo, thus improving endothelial barrier function and promoting recovery in ALI models^[5]^.

Mesenchymal stem cells (MSCs), derived from bone marrow or adipose tissue, possess self-renewal, immunomodulatory, and reparative capacities. Despite clinical promise^[6,7]^, their translation is hindered by poor homing, transient effects, cost, safety risks (immune rejection/tumorigenicity), and standardization challenges^[7]^. Functionality is microenvironment-dependent; LPS inflammation impairs survival and paracrine activity^[4]^. Combinatorial approaches (e.g., liraglutide) enhance MSC resilience via Protein Kinase A (PKA)/β-catenin signaling^[8]^.

Exosomes (30-100 nm extracellular vesicles) transport bioactive cargo (proteins, RNA) for intercellular communication^[9,10]^. MSC-Exo retain therapeutic effects as cell-free alternatives, mediating repair (anti-apoptosis, immunomodulation, angiogenesis) and serving as natural delivery vehicles^[11]^. Compared to MSCs, MSC-Exo exhibit a superior safety profile (reduced risk of immune rejection, tumorigenicity, and microvascular occlusion), stability (lipid bilayer protection), and delivery flexibility (e.g., nebulization)^[12]^, with efficacy in autoimmune diseases^[13]^, tissue repair^[14,15]^, neurodegeneration, and cancer.

MSC-Exo effectively alleviate edema and inflammation, promote epithelial repair, and modulate immunity in ALI/ARDS^[16]^. Recent studies have found that MSC-Exo show superior barrier repair compared to their parent MSCs in ARDS models^[17]^. In addition, other studies have reported that different exosomal components, including proteins, miRNAs, and lncRNAs, may regulate pulmonary vascular permeability, apoptosis, and endothelial migration, although the precise mechanisms remain incompletely understood.

Building on these findings, the present study aims to investigate the role of neuropilin-1 (NRP1) contained in MSC-Exo in regulating pulmonary microvascular endothelial cell (PMVEC) function during ALI. Specifically, we explored whether NRP1 influences endothelial apoptosis, migration, and proliferation, as well as its impact on TJ integrity and vascular permeability under LPS-induced injury. Through a combination of proteomic screening, molecular assays, and functional analyses, we sought to delineate the signaling mechanisms by which exosomal NRP1 contributes to endothelial barrier protection. By clarifying these mechanisms, our work provides novel insights into MSC-Exo-mediated vascular repair and highlights potential therapeutic strategies for ALI/ARDS.

## METHODS

### Cell culture

Bone marrow-derived mesenchymal stem cells (BMSCs, RASMX-01001) and PMVECs (BNCC359677) from rats were purchased from Cyagen Biosciences Inc. and BeNa Culture Collection (BNCC) Biotechnology Co., Ltd., respectively. BMSCs were cultured in MSC-specific medium (BLDM-03011, Cyagen Biosciences) or Complete Medium for Rat Bone Marrow MSCs (Without EXO) (RAXMX-90012, Cyagen Biosciences), PMVECs were cultured in endothelial cell-specific medium (Cat No.1001, Sicencell) with fetal bovine serum (FBS) (RAXMX-05001, Cyagen Biosciences) at 37 °C with 5% CO_2_. Routine monitoring was performed using inverted microscopy (XDS-3KY, Qibushengwu, China) guided subculturing or cryopreservation when cells once they reached 80%-90% confluence.

### Establishment of LPS-induced PMVEC injury model

PMVECs [passages 3-5 (P3-P5), logarithmic phase] at 70%-80% confluence were replenished with endothelial cell growth supplement (ECGS)-containing medium. Liraglutide was applied at a concentration of 10 nM where indicated. Cells were then allocated to four groups:

(1) Control group: Cells were cultured under standard conditions for 36 h. (2) Model group: Cells were treated with LPS (Sigma) at a final concentration of 10 μg/mL for 12 h to establish an acute inflammatory injury model, followed by an additional 24 h of culture under standard conditions. (3) Basal MSC-Exo treatment group: After LPS stimulation for 12 h, cells were treated with non-LPS-preconditioned MSC-Exo at a concentration of 200 μg/mL and further cultured for 24 h. (4) LPS-preconditioned MSC-Exo treatment group: After LPS stimulation for 12 h, cells were treated with LPS-preconditioned MSC-Exo at a concentration of 200 μg/mL and further cultured for 24 h.

### Isolation of MSC-Exo

(1) BMSCs at P3-P5 in the logarithmic growth phase were used in all experiments. When cell confluence reached approximately 70%-80%, the culture medium (MSC-specific medium, BLDM-03011, Cyagen Biosciences) was removed, and the cells were gently washed twice with sterile phosphate-buffered saline (PBS) to eliminate residual metabolites. The medium was then replaced with exosome-depleted (Exo-free) culture medium (RAXMX-90012, Cyagen Biosciences). For the LPS-preconditioning group, LPS (30 μg/mL) was added to the culture medium, whereas an equal volume of PBS was added to the control group. Cells were further incubated for 48 h.

(2) Conditioned medium was collected and sequentially centrifuged at 2,000 × *g* for 10 min at 4 °C to remove floating cells and cellular debris, followed by centrifugation at 10,000 × *g* for 30 min at 4 °C to eliminate apoptotic bodies. The resulting supernatant was either used immediately for extracellular vesicle isolation or stored at -80 °C until further processing. All centrifugation steps were performed using an Optima XPN-90 ultracentrifuge (Beckman Coulter, USA).

Exosomes were isolated from MSCs using differential ultracentrifugation as follows:

(3) Filtered supernatants (0.22 μm) underwent centrifugation at 120,000 × *g* for 90 min at 4 °C. Supernatants were carefully aspirated, retaining 5 mL concentrated pellets.

(4) Pellets were resuspended in pre-chilled PBS, transferred to ultracentrifuge tubes, and centrifuged at 120,000 × *g* for 70 min at 4 °C. Final pellets were resuspended in pre-chilled PBS and stored at -80 °C.

### Exosome treatment

Exosomes were quantified using a bicinchoninic acid (BCA) assay. Equal amounts of exosomes (10 μg per well for *in vitro* experiments) were added to the cell culture medium unless otherwise specified.

### Transmission electron microscopy analysis of extracellular vesicles

Extracellular vesicle morphology was examined by transmission electron microscopy (TEM, HT-7700, Hitachi). Briefly, carbon-coated copper grids were placed on sterile filter paper, and 10 μL of extracellular vesicle suspension was applied onto the grid surface and allowed to adsorb for 1 min. Excess liquid was gently removed from the edge of the grid at an angle of approximately 45° using filter paper.

Negative staining was performed by adding 10 μL of 2% (w/v) uranyl acetate solution (pH 4.5) onto the grid, followed by incubation for 1 min in the dark. Excess stain was removed in the same manner. The grids were then air-dried at room temperature for 10 min.

TEM images were acquired using a transmission electron microscope operated at an accelerating voltage of 100 kV, and representative images were recorded to visualize the ultrastructural features of extracellular vesicles.

### Western blot analysis of MSC-Exo marker and contaminant proteins

Western blotting was performed to assess the expression of MSC-Exo-specific marker proteins CD9 and TSG101, as well as mitochondrial contaminant protein CYC1 and endoplasmic reticulum contaminant protein GRP94.

Preparation of cellular protein lysates: Cells were washed twice with prechilled PBS and lysed on ice using Radioimmunoprecipitation Assay (RIPA) buffer (PC101, Epizyme, China) supplemented with protease inhibitors, dithiothreitol (DTT) (D8220, Solarbio), and phenylmethylsulfonyl fluoride (PMSF) (P8340, Solarbio). Lysates were incubated for 20 min and subsequently centrifuged at 13,000 × *g* for 25 min at 4 °C. The supernatants were collected and stored on ice for further analysis.

Protein concentrations were determined using a BCA assay according to the manufacturer’s instructions. Equal amounts of protein were mixed with 5× sodium dodecyl sulfate (SDS) loading buffer and denatured at 95 °C for 15 min.

Preparation of MSC-Exo protein samples: Protein concentrations of MSC-Exo suspensions were determined by BCA assay. Samples were diluted with PBS to match the protein concentration of cellular lysates, mixed with 5× SDS loading buffer, and denatured at 95 °C for 15 min.

Sodium dodecyl sulfate–polyacrylamide gel electrophoresis (SDS-PAGE) and Western blot: Proteins were separated by SDS-PAGE and transferred to PVDF membranes (0.45 μm Millipore). PVDF membranes were activated in 100% methanol for 40 s, equilibrated in transfer buffer, and assembled in a standard Bio-Rad transfer sandwich. Proteins were transferred at a constant current of 400 mA for 30-40 min depending on molecular weight.

Membranes were blocked with blocking buffer for 20 min at room temperature and washed with 1× Tris-buffered saline with Tween 20 (TBST). Membranes were incubated overnight at 4 °C with primary antibodies against CD9, TSG101, CYC1, or GRP94, diluted according to the manufacturers’ instructions (1:1,000). After washing, membranes were incubated with horseradish peroxidase (HRP)-conjugated secondary antibodies in 5% BSA/TBST for 1.5 h at room temperature.

Protein bands were visualized using enhanced chemiluminescence (ECL) reagents. Images were captured using a chemiluminescence imaging system, and band intensities were quantified using ImageJ software.

### Flow cytometry analysis of MSC-Exo surface markers CD63 and CD81

Flow cytometry was used to verify the expression of MSC-Exo-specific markers CD63 and CD81.

Exosome labeling: Fifty microliters of MSC-Exo suspension were aliquoted into flow cytometry tubes. Experimental groups were incubated with 1 μL CD63/CD81-FITC antibody, isotype control groups with 1 μL FITC-IgG1, and blank control groups with 1 μL PBS. Tubes were gently vortexed and incubated at 4 °C in the dark for 1 h.

Antibody washing: One milliliter of PBS containing 1% BSA was added to each tube. Tubes were centrifuged to remove unbound antibodies. After two washes, the labeled MSC-Exo were resuspended in 100 μL PBS.

Flow cytometry acquisition: Data were acquired using CytoFLEX LX and CytoFLEX S (Beckman) equipped with 488 nm laser and 525/40 nm emission filter at low flow rate, with 10,000 events recorded per sample. Data analysis was performed using FlowJo software.

### Flow cytometry analysis of the effect of MSC-Exo on LPS-induced apoptosis in PMVECs

Annexin V-FITC/propidium iodide (PI) double staining was used to detect the effect of MSC-Exo on reducing LPS-induced apoptosis in PMVECs. The procedure was as follows:

Cell preparation: Cells were treated according to the grouping described in section “Establishment of LPS-Induced PMVEC Injury Model”. After digestion with 0.25% trypsin, the reaction was stopped immediately by adding medium containing 10% FBS. Cells were collected by centrifugation at 2,000 × *g* for 5 min at 4 °C, washed once with cold PBS under the same conditions, and finally resuspended in 300 μL cold 1× binding buffer.

Cell staining: Blank controls (unstained) and single-stained controls (Annexin V-FITC or PI only) were set up. Five microliters of Annexin V-FITC were added to the experimental samples, gently mixed, and incubated at room temperature in the dark for 15 min. Prior to acquisition, 5 μL PI was added, and the volume was adjusted with 200 μL binding buffer.

Flow cytometry acquisition: FITC and PI channels were used to detect stained cells. Data were acquired for 10,000 events per sample, and apoptosis rates were analyzed using FlowJo software.

### Transwell migration assay

PMVECs (80%-90% confluent) were serum-starved for 24 h, trypsinized with 0.25% trypsin containing ethylenediaminetetraacetic acid (EDTA), and centrifuged (1,000 × *g*, 3 min). After PBS washes, cells were resuspended in serum-free medium at 1 × 10^5^ cells/mL.

A total of 200 μL of cell suspension was seeded into the upper chamber of Transwell inserts (8 μm pore size). Exosomes (10 μg per well) were added to the upper chamber together with the cells. The lower chamber was filled with 600 μL of medium containing 10% FBS as a chemoattractant. Inserts were positioned in 24-well plates and incubated for 24 h.

Post-incubation, cells were fixed with 4% paraformaldehyde (PFA) for 30 min, stained with 0.1% crystal violet for 20 min) and washed with distilled water. Migrated cells in five random fields/insert were counted via ImageJ after imaging.

### Colony formation assay

A matrix-free colony formation assay was performed to evaluate the clonogenic capacity of cells.

Cell seeding: Cells were seeded into six-well plates at a density of approximately 500-1,000 cells per well in 2 mL of complete culture medium. Plates were gently rocked to ensure even cell distribution. Cells were treated as described in Section 4.2.3 and incubated at 37 °C with 5% CO_2_ for 1-2 weeks until visible colonies formed.

Fixation: The medium was removed, and cells were washed twice with PBS. Colonies were fixed with 1 mL of 4% PFA per well at room temperature for 15 min.

Staining: After fixation, wells were washed twice with PBS and stained with 1 mL crystal violet solution per well at room temperature for 15 min. Excess dye was gently rinsed off with running water until the background was clear. Plates were air-dried at room temperature.

Quantitative analysis: Colony formation was observed under natural light and photographed. The percentage of colony area (Area%) was quantified using ImageJ software.

### Scratch wound healing assay

PMVECs were seeded in 6-well plates (5 × 10^5^ cells/well). After 12 h LPS treatment (10 μg/mL), uniform wounds (~0.5-1 mm width) were created using sterile pipette tips. Wells were washed with PBS, replenished with serum-free medium containing MSC-Exo (200 μg/mL), and incubated for 24 h. Wound closure was quantified at 0/24 h via microscopy and ImageJ-based migration rate (%) analysis.

### Protein extraction and quantification

MSCs were treated with 30 μg/mL LPS ± 10 nM liraglutide in 6-well plates for 48 h. Media supernatants were collected and lysed in SDS-Tris buffer (4% SDS, 100 mM Tris-HCl, pH 7.6) followed by centrifugation (14,000 × *g*, 15 min).

Protein concentration was determined via endogenous tryptophan fluorescence (excitation: 295 nm, emission: 350 nm). Tryptophan standards (0-200 ng/μL) prepared in SDT buffer were diluted in urea buffer (8 M urea, 20 mM Tris/HCl, pH 7.6). Sample tryptophan content was calculated from the standard curve, with total protein derived by dividing values by 1.3% (tryptophan-to-protein ratio).

### Filter-aided sample preparation (FASP) digestion

Protein samples (200 μg) were denatured in urea buffer (UA buffer) (8 M urea, 150 mM Tris-HCl, pH 8.5) and loaded onto 30 kDa centrifugal filters. After centrifugation (14,000 × *g*, 15 min × 2), alkylation was performed with 50 mM iodoacetamide in UA buffer for 30 min in the dark at room temperature (RT). Following UA buffer washes, buffer exchange used 50 mM ammonium bicarbonate (ABC).

Trypsin digestion (5 μg in 40 μL ABC) proceeded for 18 h at 37 °C. Peptides were collected by centrifugation (14,000 × *g*, 10 min), acidified with 0.1% trifluoroacetic acid (TFA), and desalted using C18 StageTips per manufacturer’s protocols.

### Mass spectrometry analysis

Peptides (5 μg) were separated via nanoLC using a 240-min gradient at 300 nL/min: mobile phase A (0.1% formic acid in H_2_O) and B (0.1% formic acid in acetonitrile). The gradient progressed from 0% to 45% B (0-200 min), 45% to 100% B (200-216 min), and 100% B (216-240 min).

MS analysis was performed in positive ion mode with a 1.8 kV spray voltage and m/z 350-1,800 scan range. Full-scan MS1 spectra were acquired at 60,000 resolution (m/z 400), followed by data-dependent MS2 scans using ion trap collision-induced dissociation (CID) fragmentation (normalized collision energy 35%, *q*-value 0.25, 30 ms activation time) in centroid mode. Dynamic exclusion was set to 30 s. Instrument calibration was performed prior to acquisition.

Instrument: Q Exactive HF-X (Thermo Fisher Scientific); Data processing: MaxQuant v2.1.0.

### Label-free quantitative analysis by MaxQuant

MS raw files were processed using MaxQuant (v1.6.17.0) with the Andromeda search engine against the UniProt Rattus norvegicus database (release 2024_05; 92,948 entries). False discovery rates (FDR) for peptides and proteins were determined via reverse decoy database.

Label-free quantification (LFQ) was performed using MaxQuant’s built-in algorithm, which integrates isotopic peak intensities across runs. Proteins identified exclusively by modification sites, reverse matches, or common contaminants were excluded.

### Bioinformatics analysis

Differentially expressed proteins (DEPs) were defined as proteins exhibiting a ≥ 1.2-fold change and *P* < 0.05 (detected in ≥ 50% of replicates). All DEPs underwent functional annotation via Blast2GO, with Gene Ontology (GO) enrichment analysis across molecular functions, cellular components, and biological processes. Kyoto Encyclopedia of Genes and Genomes (KEGG) pathway enrichment identified associations with metabolism, signal transduction, and disease-related pathways. Fisher’s exact test (*P* < 0.05) determined statistical significance for all enrichments.

### Plasmid and siRNA transfection

Plasmid transfection: Plasmids were purchased from Shanghai GenePharma Co., Ltd. (Shanghai, China). MSCs were seeded into six-well plates in advance, and transfection was performed when cell confluence reached 70%-80%. The culture medium was replaced immediately before transfection.For each transfection, 125 μL serum-free, antibiotic-free Dulbecco’s Modified Eagle Medium (DMEM), 2.5 μg plasmid DNA, and 4 μL Lipo8000 transfection reagent were sequentially added into a sterile microcentrifuge tube and gently mixed. A total of 125 μL of the transfection mixture was slowly added to each well. Cells transfected with empty vector served as the negative control.After 48 h of incubation, cell culture supernatants were collected for MSC-Exo isolation.

siRNA transfection: siRNAs were also purchased from Shanghai GenePharma Co., Ltd. (Shanghai, China). The cell preparation and transfection procedures were identical to those used for plasmid transfection.The transfection mixture was prepared by sequentially adding 125 μL serum-free, antibiotic-free DMEM, 100 pmol siRNA, and 4 μL Lipo8000 transfection reagent, followed by gentle mixing and incubation at room temperature for 20 min. The mixture was then added to the cells.Cells transfected with scrambled siRNA were used as the negative control. The siRNA sequences are listed in [Table 1].

**Table 1:** Sequences of siRNAs used in this study

**Code**	**Sequence**
rNRP1 si-1	F: 5’-CACCCUGAAGGAGGGAAAUAATT-3’ R: 5’-UUAUUUCCCUCCUUCAGGGUGTT-3’
rNRP1 si-2	F: 5’-GCAUGGUGUCUGGACUUAUUUTT-3’ R: 5’-AAAUAAGUCCAGACACCAUGCTT-3’
rNRP1 si-3	F: 5’-CCGAAUGUUCUCAGAACUAUATT-3’ R: 5’-UAUAGUUCUGAGAACAUUCGGTT-3’

siRNA: Small interfering RNA; NRP1: neuropilin-1; rNRP1: rat neuropilin-1.

### Immunofluorescence staining

MSCs were seeded in confocal dishes and transfected at 50%-70% confluency. After 48 h, cells were fixed with 4% PFA (25 min, RT), permeabilized with 2% Triton X-100/PBS (10 min, RT), and blocked with 4% BSA/PBS (1 h, RT). Primary antibodies (diluted in 4% BSA/PBS) were incubated overnight at 4 °C. Following PBS washes, secondary antibodies (4% BSA/PBS) were applied for 2 h (RT, dark). Nuclei were counterstained with DAPI (5 min, RT). Images were acquired using a confocal microscope and analyzed with ImageJ.

### Establishment of LPS-induced ALI rat model

Thirty-two male Sprague-Dawley rats (5-6 weeks, 220 ± 20 g; Vital River Laboratory, Beijing) were acclimatized for one week under SPF conditions and randomly divided into four groups (*n* = 8/group):

Control: 100 μL PBS intratracheally; ALI: 5 mg/kg LPS (O55:B5, Sigma #L2880) in 100 μL PBS; Control-Exo: LPS + 200 μg basal MSC-Exo; OE-Exo: LPS + 200 μg NRP1-overexpressing MSC-Exo. Rat experiments were reviewed and approved by Ruijin Hospital Ethics Committee (approval No. RJ2025030).

### Evans blue staining

Vascular permeability was assessed via Evans blue (EB) extravasation. Rats received tail vein injections of EB solution (10 mg/mL in saline, 5 mL/kg). After 60 min, lungs were harvested following CO_2_ euthanasia. Tissues were homogenized and incubated in formamide at 65 °C for 24 h to extract dye. EB concentration in supernatants was quantified by OD_620_ measurement using a microplate reader.

### BCA assay for bronchoalveolar lavage fluid total protein concentration

Following euthanasia, bronchoalveolar lavage fluid (BALF) was collected by cannulating the left main bronchus after ligating the right bronchus. Ice-cold saline (3 mL × 3) was slowly instilled and aspirated. BALF supernatants were obtained by centrifugation (2,000 × *g*, 10 min, 4 °C). Total protein concentration was determined via BCA assay (Pierce^TM^) after serial dilution within the standard curve range (BSA standards).

### HE staining and histopathological scoring of rat lung tissues

Lung tissues were fixed in 4% neutral-buffered formalin (24 h), processed through graded ethanol dehydration (75%→100%), cleared in xylene, and embedded in paraffin. Sections (4-5 μm) were stained with hematoxylin and eosin (H&E).

Histopathological scoring (0-4 scale) assessed alveolar hemorrhage, neutrophil infiltration, and septal thickening across five random fields/section by blinded pathologists, per established lung injury criteria.

### Lung wet/dry weight ratio measurement

The right middle lung lobe was excised immediately post-euthanasia, blotted dry, and weighed (wet weight). Tissues were dehydrated at 65 °C for 48 h to constant weight (dry weight). The wet-to-dry weight ratio (W/D ratio) was calculated as W_wet_/W_dry_.

### Immunohistochemical staining of lung tissues

Paraffin sections underwent deparaffinization in xylene and rehydration through graded ethanol. Antigen retrieval was performed in Tris-EDTA buffer (pH 9.0) using heat-induced epitope retrieval (15 min boiling followed by 15 min at 37 °C). Endogenous peroxidase activity was quenched with 3% H_2_O_2_ (10 min, RT).

After blocking with 5% BSA (30 min, RT), sections were incubated with primary antibodies against OCLN/ZO-1 overnight at 4 °C in a humidified chamber. HRP-conjugated secondary antibodies were applied (45 min, 37 °C), followed by 3,3'-diaminobenzidine (DAB) development monitored microscopically for brown precipitate formation. Counterstaining used hematoxylin with bluing in running water.

Quantitative analysis determined the positive signal area ratio (brown DAB staining *vs*. total tissue area) using ImageJ immunohistochemistry (IHC) Profiler.

### Antibody information

CD63 (FITC) Antibody (Ab108949), FITC-conjugated mouse IgG1 isotype control (Ab91356), NRP1 Antibody (Ab81321), MMP9 Antibody (Ab76003), platelet-derived growth factor receptor alpha (PDGFR-α) Antibody (Ab203491), Alexa Fluor® 555-conjugated goat anti-rabbit IgG (Ab150078), Alexa Fluor® 488-conjugated goat anti-rabbit IgG (Ab150077) were purchased from Abcam. CD81 (FITC) Antibody (Sc-23962) were purchased from Santa Cruz Biotechnology. CYC1 Antibody (DF4698), B-cell lymphoma 2 (Bcl-2) Antibody (AF6139), Phospho-ERK1/2 Antibody (AF1015), cytochrome c (Cyt c) Antibody (AF0146) were purchased from Affinity. GRP94 Antibody (14700-1-AP), CD9 Antibody (20597-1-AP), TSG101 Antibody (28283-1-AP), HRP-conjugated goat anti-rabbit IgG secondary antibody (Sa00001-2), β-actin Antibody (20536-1-AP), MMP1 Antibody (10371-2-AP), p130Cas Antibody (16815-1-AP), Bax Antibody (50599-2-Ig), Cleaved Caspase3 Antibody (25128-1-AP), Occludin Antibody (13049-1-AP), ZO-1 Antibody (21773-1-AP) were purchased from Proteintech (Wuhan, China). Cleaved Caspase9 Antibody (Cy5682) was purchased from Abways. p-p130Cas Antibody (13653) was purchased from Sabbiotech.

### Statistical analysis

All experiments were performed with three independent biological replicates. Data are presented as mean ± standard deviation (SD). Statistical comparisons among multiple groups were performed using one-way analysis of variance (ANOVA) followed by appropriate post hoc multiple comparison tests in GraphPad Prism 9.5. For comparisons between two groups, two-tailed Student’s *t*-test was used where applicable. Statistical significance was defined as ^*^P < 0.05, ^**^P < 0.01, ^***^P < 0.001.

All figures, including the graphical abstract, were created by the authors. Graphical and statistical analyses were performed using GraphPad Prism 9.5, FlowJo, and ImageJ. Schematic diagrams were prepared using Adobe Illustrator.

## RESULTS

### Characterization of isolated exosomes

TEM was used to visualize vesicle-like structures in the isolated MSC-Exo preparations, revealing membrane-bound particles with sizes consistent with small extracellular vesicles (Supplementary Figure 1A; scale bar = 200 nm). Western blot analysis demonstrated enrichment of exosomal markers CD9 and TSG101 in MSC-Exo concentrates compared with whole-cell lysates, while the endoplasmic reticulum protein GRP94 and mitochondrial protein CYC1 were not detectable, indicating minimal cellular contamination [Supplementary Figure 1B]. Nanoparticle tracking analysis (NTA) further confirmed a relatively homogeneous size distribution of MSC-Exo, with particle diameters predominantly within the typical extracellular vesicle range [Supplementary Figure 1C]. Flow cytometry also confirmed the high purity of MSC-Exo preparations, with 89.4% of vesicles positive for CD63 and 99.1% positive for CD81 [Supplementary Figure 1D]. Collectively, these results support the successful isolation of MSC-derived extracellular vesicles enriched in commonly used exosomal markers, with low levels of detectable cellular protein contamination, suitable for downstream functional assays.

### MSC-Exo attenuated LPS-induced endothelial cell apoptosis and promoted migration

**Figure 1:**
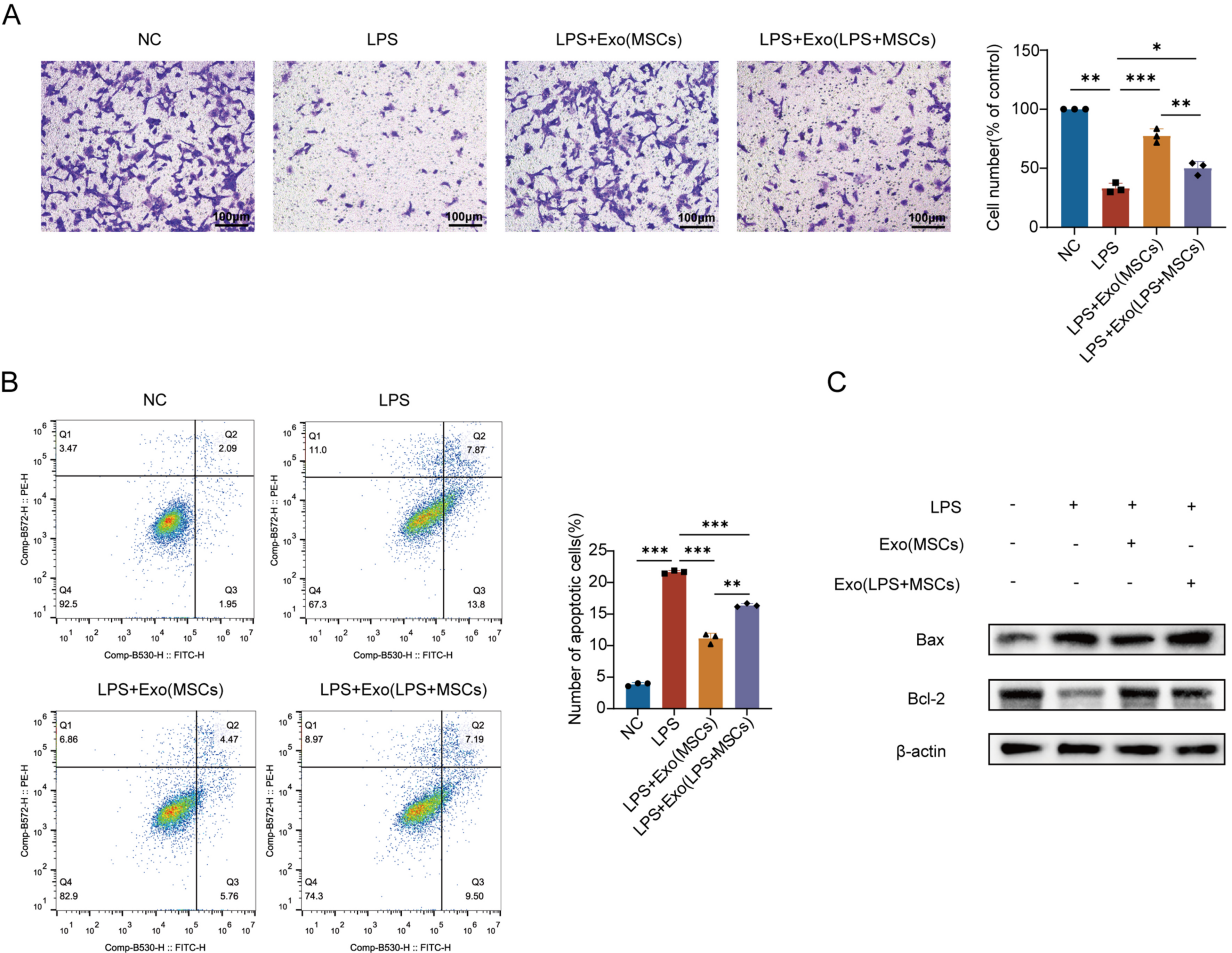
Characterization of MSC-Exo and their effects on LPS-induced endothelial migration dysfunction and apoptosis. (A) Effects of MSC-Exo collected under different conditions on LPS-induced migration dysfunction in PMVECs. Quantitative analysis of migrated cells. Scale bar = 50 μm. The experiment was independently repeated three times; (B) Protective effects of MSC-Exo collected under different conditions on LPS-induced apoptosis in PMVECs. Quantitative analysis of apoptosis rate; (C) Western blot detection of Bcl-2 and Bax protein expression in PMVECs treated with MSC-Exo. The experiment was independently repeated three times. Data are presented as mean ± SD. Statistical analysis was performed using one-way ANOVA followed by Tukey’s multiple comparisons test. ^*^P < 0.05; ^**^P < 0.01; ^***^P < 0.001. MSC: Mesenchymal stem cell; MSC-Exo: mesenchymal stem cell-derived exosomes; Exo: exosome; LPS: lipopolysaccharide; PMVECs: pulmonary microvascular endothelial cells; NC: normal control; Exo (MSCs): exosomes derived from mesenchymal stem cells; Exo (LPS + MSCs): exosomes derived from lipopolysaccharide-treated mesenchymal stem cells; Bcl-2: B-cell lymphoma 2; Bax: Bcl-2-associated X protein; SD: standard deviation; ANOVA: analysis of variance.

We evaluated the migratory capacity of PMVECs under different conditions. LPS stimulation (10 μg/mL) significantly impaired PMVECs migration, reducing the number of transmigrated cells by 66.8% (*P* < 0.01) *vs*. normal controls with dense crystal violet-stained membranes. Both basal and LPS-pretreated MSC-Exo (200 μg/mL) partially restored migration in LPS-injured PMVECs, enhancing chemoattractant response. However, LPS-pretreated MSC-Exo showed markedly limited efficacy compared to basal MSC-Exo, with migration rates markedly lower than normal levels (*P* < 0.05) and significantly inferior to basal MSC-Exo treatment (*P* < 0.01), indicating LPS preconditioning substantially compromises MSC-Exo’s promigratory function [Figure 1A].

To assess the impact of LPS-preconditioned exosomes on endothelial apoptosis, Annexin V-FITC/PI dual staining revealed significantly elevated apoptosis rates in LPS-injured PMVECs (an absolute increase of 17.8% compared to normal controls; *P* < 0.01), indicating LPS-induced disruption of cellular homeostasis. Treatment with either basal or LPS-preconditioned MSC-Exo (200 μg/mL) partially attenuated apoptosis in injured PMVECs, though LPS-preconditioned exosomes exhibited reduced efficacy [Figure 1B].

However, while LPS-preconditioned MSC-Exo reduced apoptosis *vs*. LPS-injured controls (*P* < 0.05), its anti-apoptotic efficacy was 32.0% lower than basal exosomes (*P* < 0.01). Western blot analysis confirmed attenuated downregulation of pro-apoptotic Bax and impaired recovery of anti-apoptotic Bcl-2 in the LPS-preconditioned exosome group *vs*. basal treatment. Collectively, these results demonstrate that LPS preconditioning compromises the endothelial protective capacity of MSC-Exo [Figure 1C].

### Liraglutide promoted the therapeutic effect of MSCs exosomes and increased the expression of NRP1 in exosomes

**Figure 2:**
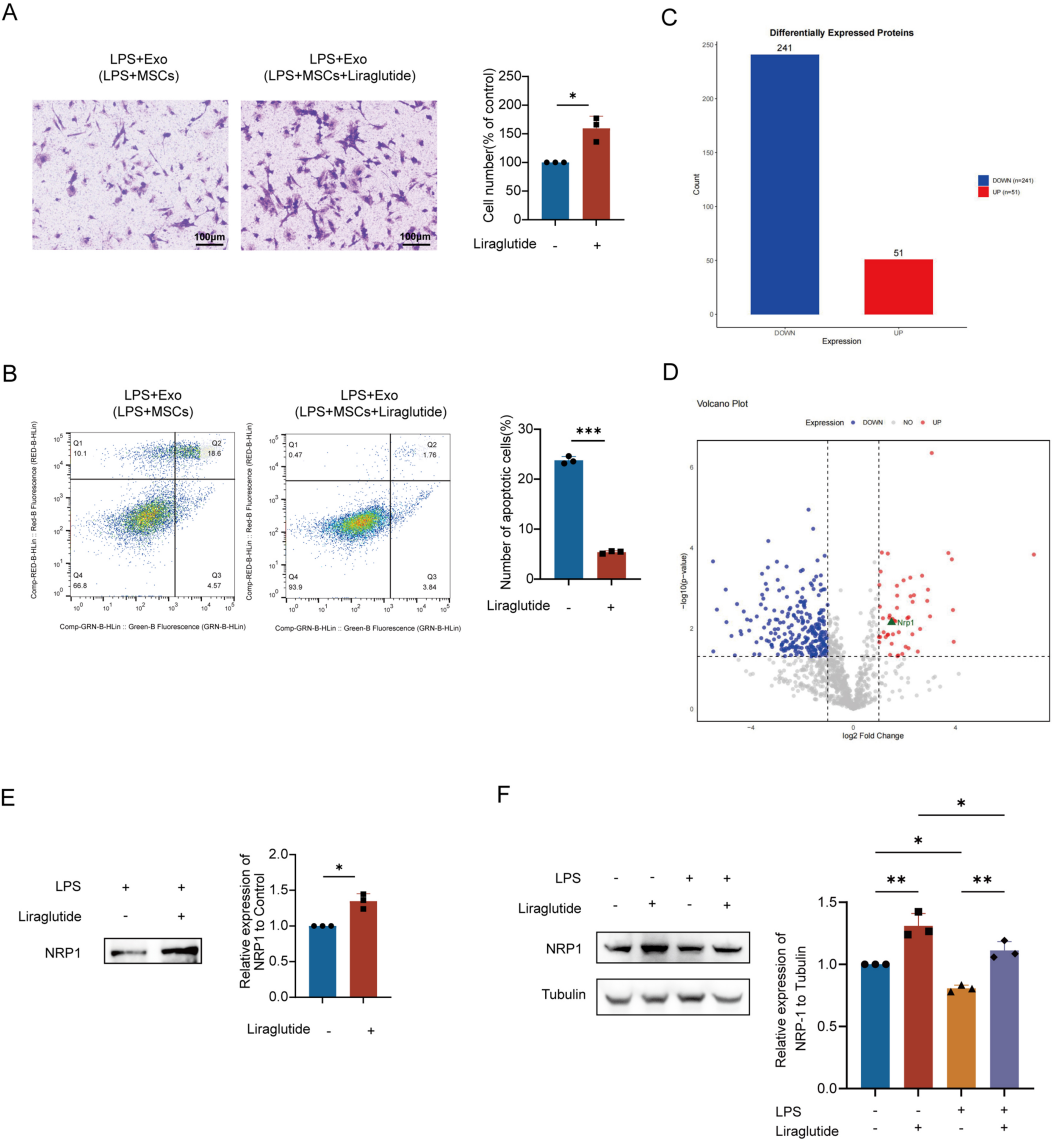
Liraglutide promoted the therapeutic effect of MSCs exosomes and increased the expression of NRP1 in exosomes. (A) Transwell assay demonstrates that liraglutide enhanced the LPS-pretreated MSC-Exo-mediated migration of PMVECs. Scale bar = 50 μm; (B) Annexin V-FITC/PI flow cytometry confirms that liraglutide strengthened the LPS-pretreated MSC-Exo-induced suppression of PMVEC apoptosis. All experiments were independently repeated three times; (C) Bar plot showing quantitative distribution of up-/down-regulated proteins (X-axis: regulation direction; Y-axis: protein count); (D) Volcano plot visualizing differential expression [X-axis: log2(fold change) with left/right quadrants indicating down-/up-regulation; Y-axis: -log10(*P*-value)]. Distance from origin correlates with statistical significance; (E) Combination treatment with liraglutide and LPS for 48 h significantly upregulated NRP1 protein levels in MSC-Exo. Equal amounts of exosomal protein (10 μg per lane) were loaded based on BCA quantification; (F) NRP1 expression in PMVECs under different treatments (Control, LPS, Liraglutide, and Liraglutide + LPS). LPS stimulation (12 h) resulted in a significant reduction in NRP1 expression, while liraglutide partially reversed this effect. Liraglutide (10 nM) was applied as indicated. All experiments were independently repeated three times. Data are presented as mean ± SD. Statistical analysis for (A, B, and E) was performed using unpaired Student’s *t*-test, while (F) was analyzed using one-way ANOVA followed by Tukey’s multiple comparisons test. ^*^P < 0.05; ^**^P < 0.01; ^***^P < 0.001. LPS: Lipopolysaccharide; Exo: exosome; MSCs: mesenchymal stem cells; MSC-Exo: mesenchymal stem cell-derived exosomes; Exo (LPS + MSCs): exosomes derived from lipopolysaccharide-treated mesenchymal stem cells; PMVECs: pulmonary microvascular endothelial cells; NRP1: neuropilin-1; FITC: fluorescein isothiocyanate; PI: propidium iodide; BCA: bicinchoninic acid; SD: standard deviation; ANOVA: analysis of variance.

Liraglutide, a glucagon-like peptide-1 (GLP-1) analog, suppresses endothelial-mesenchymal transition and macrophage inflammation. Our prior work demonstrated its synergy with MSCs in ALI treatment: liraglutide inhibited LPS-induced MSC apoptosis while enhancing secretion of vascular repair factors (Ang-1), surfactant protein C (SPC), and keratinocyte growth factor (KGF), outperforming monotherapies^[8]^. We therefore assessed whether liraglutide could rescue the impaired repair capacity of LPS-preconditioned MSC-Exo on PMVECs. Transwell assays [Figure 2A] revealed that LPS-preconditioned MSC-Exo (control) failed to rescue LPS-induced migration dysfunction, whereas exosomes collected from MSCs co-treated with liraglutide and LPS (liraglutide + LPS-preconditioned MSC-Exo)significantly enhanced migration (1.6-fold increase in crystal violet-positive cells *vs*. control; *P* < 0.05). Similarly, flow cytometry [Figure 2B] demonstrated reduced apoptosis in the liraglutide + LPS group (18.4% decrease *vs*. control; *P* < 0.001), indicating restored cellular viability. These results establish liraglutide’s efficacy in reversing LPS-mediated impairment of exosomal repair functions.

MSCs were divided into experimental (10 nM liraglutide + 30 μg/mL LPS) and control (LPS-only) groups. Proteomic analysis of 48 h-conditioned supernatants via LC-MS/MS with LFQ identified DEPs mapped against the UniProt rat database. GO enrichment [Supplementary Figure 2A] revealed DEPs in liraglutide-preconditioned exosomes were enriched in energy metabolism, regenerative repair, and oxidative stress response. Complementary KEGG analysis [Supplementary Figure 2B] demonstrated significant associations with endothelial cytoskeletal dynamic pathways, energy biosynthesis pathway, and epithelial barrier restoration pathways. These findings suggest that liraglutide-preconditioned exosomes enhance endothelial repair through dual mechanisms: promoting energy-driven cell migration/proliferation while regulating cytoskeletal remodeling for barrier integrity.

Proteomic analysis of liraglutide + LPS-preconditioned MSC-Exo identified 292 DEPs, with 51 significantly upregulated [fold change (FC) ≥ 2, *P* < 0.05] and 241 downregulated (FC ≤ 0.5, *P* < 0.05). The complete list of DEPs is provided in Supplementary Table 1. Among upregulated DEPs, NRP1, a regulator of vascular endothelial function, showed increased expression with a fold change of 2.82 (*P* = 0.007) [Figure 2C and D]. These findings suggest that NRP1 may mediate liraglutide-induced enhancement of exosomal endothelial repair functions.

Western blotting validated the proteomic findings, demonstrating significantly elevated NRP1 expression in liraglutide + LPS-preconditioned MSC-Exo compared to LPS-only controls [Figure 2E]. Furthermore, LPS stimulation (12 h) resulted in a significant decrease in NRP1 expression in PMVECs, whereas liraglutide treatment partially restored NRP1 levels under LPS conditions. Liraglutide alone did not significantly alter NRP1 expression compared with the control [Figure 2F]. These results demonstrate that liraglutide preconditioning enhances the functional effects of MSC-Exo and is associated with increased NRP1 enrichment within, exosomes, contributing to improved endothelial cell migration and survival under LPS-induced injury conditions.

### Effect of up-regulation of NRP1 level in MSC-Exo on vascular endothelial repair

Endothelial repair involves coordinated inhibition of apoptosis, proliferation, and migration. Injured endothelial cells can proliferate and migrate to restore barrier function, while neovascularization improves perfusion and accelerates recovery.

**Figure 3:**
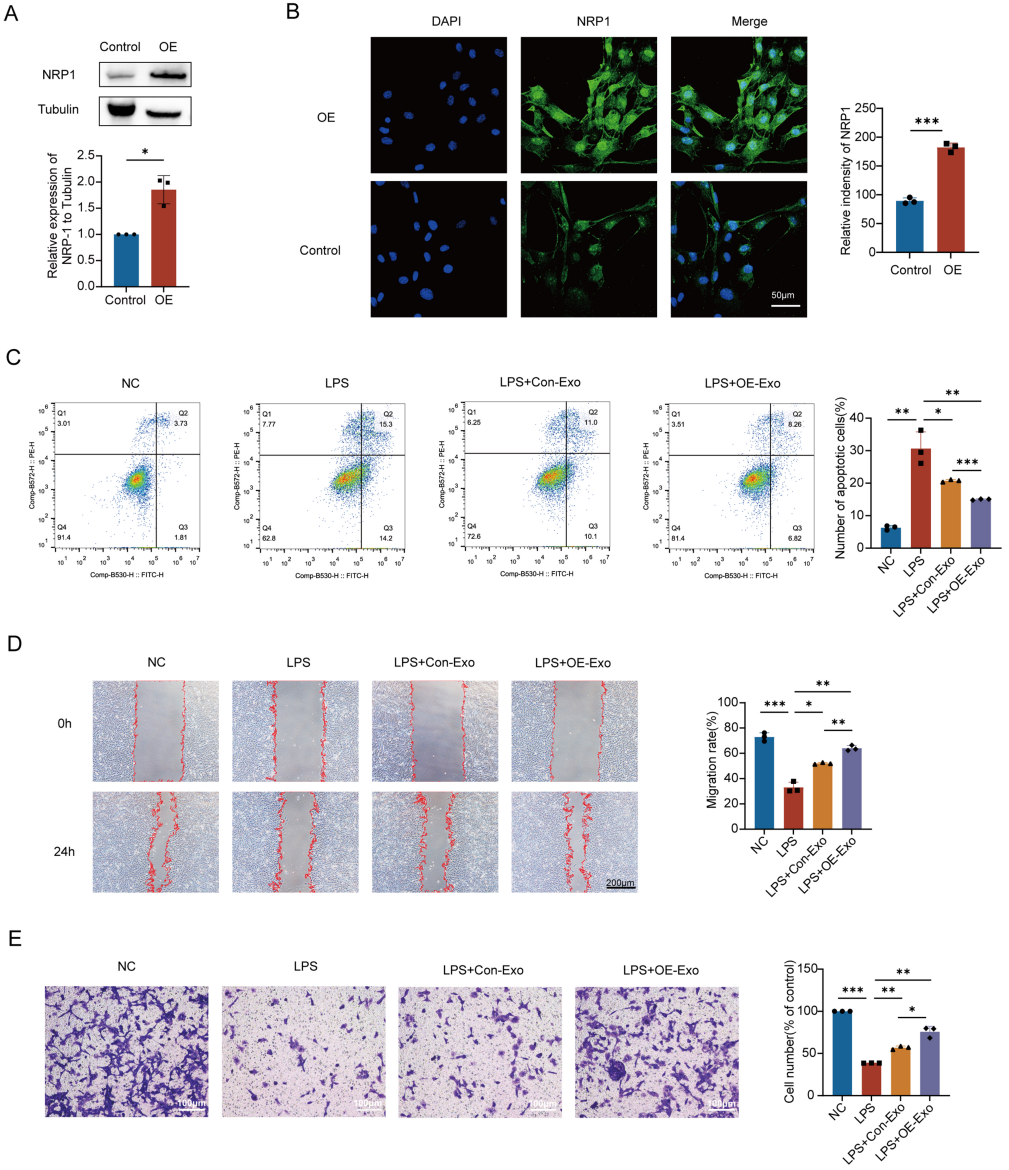
Effect of up-regulation of NRP1 level in MSC-Exo on vascular endothelial repair. (A) Western blot analysis confirmed that NRP1 protein levels were significantly increased in plasmid-transfected MSCs compared to controls; (B) Immunofluorescence analysis demonstrated a significant upregulation of NRP1 expression in MSCs transfected with the overexpression plasmid; (C) Annexin V-FITC/PI assay demonstrated that NRP1-enriched MSC-Exo significantly suppressed apoptosis in LPS-injured PMVECs; (D) Wound healing assay demonstrated the pro-migratory effect of NRP1-overexpressing MSC-Exo on injured PMVECs; (E) Transwell migration assay confirms the enhanced pro-migratory effect mediated by NRP1-overexpressing MSC-Exo. Scale bar = 50 μm. All experiments were independently repeated three times. Data are presented as mean ± SD. Statistical analysis for (A and B) was performed using unpaired Student’s *t*-test, while (C-E) were analyzed using one-way ANOVA followed by Tukey’s multiple comparisons test. ^*^P < 0.05; ^**^P < 0.01; ^***^P < 0.001. NRP1: Neuropilin-1; MSCs: mesenchymal stem cells; MSC-Exo: mesenchymal stem cell-derived exosomes; OE: overexpression; OE-Exo: exosomes derived from NRP1-overexpressing mesenchymal stem cells; Con-Exo: exosomes derived from control mesenchymal stem cells; LPS: lipopolysaccharide; PMVECs: pulmonary microvascular endothelial cells; FITC: fluorescein isothiocyanate; PI: propidium iodide; DAPI: 4’,6-diamidino-2-phenylindole; NC: normal control; SD: standard deviation; ANOVA: analysis of variance.

To clarify the independent regulatory role of NRP1 in MSC-Exo on endothelial repair, we transfected MSCs with an NRP1 overexpression plasmid or siNRP1 and verified the efficiency using immunofluorescence and Western blot. It showed that NRP1 protein expression in MSCs was significantly increased in the overexpression group compared to the empty vector group [Figure 3A]. Fluorescence imaging at 48 h post-transfection showed significantly stronger green fluorescence in the overexpression group, indicating successful upregulation of NRP1 [Figure 3B]. Western blot analysis further confirmed that NRP1 expression was significantly increased in exosomes derived from NRP1-overexpressing MSCs compared with empty vector controls [Supplementary Figure 3A], indicating successful packaging of NRP1 into MSC-Exo following transfection. Consistently, NRP1 levels were reduced in exosomes derived from siNRP1-transfected MSCs.

Flow cytometry analysis showed that LPS stimulation increased the apoptosis rate of PMVECs by approximately 24.3% compared to the normal control group. Treatment with MSC-Exo from both NRP1-overexpressing and empty vector MSCs reduced LPS-induced apoptosis to varying degrees, with the NRP1-overexpressing MSC-Exo group showing a stronger anti-apoptotic effect - reducing apoptosis by about 15.6% compared to the LPS group and by 5.8% relative to the empty vector group [Figure 3C]. In addition, the colony formation assay demonstrated that normal PMVECs formed numerous dense purple colonies, while LPS stimulation reduced colony area by approximately 79.1%. Treatment with NRP1-overexpressing MSC-Exo significantly promoted proliferation, with colony area nearly doubling compared to the empty vector group and approaching physiological levels [Supplementary Figure 3B]. These results indicate that upregulation of NRP1 enhances the ability of MSC-Exo to suppress apoptosis and promote proliferation in injured PMVECs.

Scratch wound assay showed that normal PMVECs had strong migratory ability, with nearly complete wound closure after 24 h, while LPS significantly impaired migration, leaving clear wound edges and reduced migration area. Both NRP1-overexpressing and empty vector MSC-Exo partially restored migration, with the NRP1-overexpressing group showing significantly greater improvement [Figure 3D]. Transwell assay further confirmed this effect: LPS markedly reduced the number of migrated, crystal violet-positive cells, while NRP1-overexpressing MSC-Exo significantly restored migration, surpassing the empty vector group [Figure 3E].

These results suggest that NRP1-enriched MSC-Exo exhibit enhanced protective effects on injured PMVECs.

### Effect of down-regulation of NRP1 level in MSC-Exo on vascular endothelial repair

**Figure 4:**
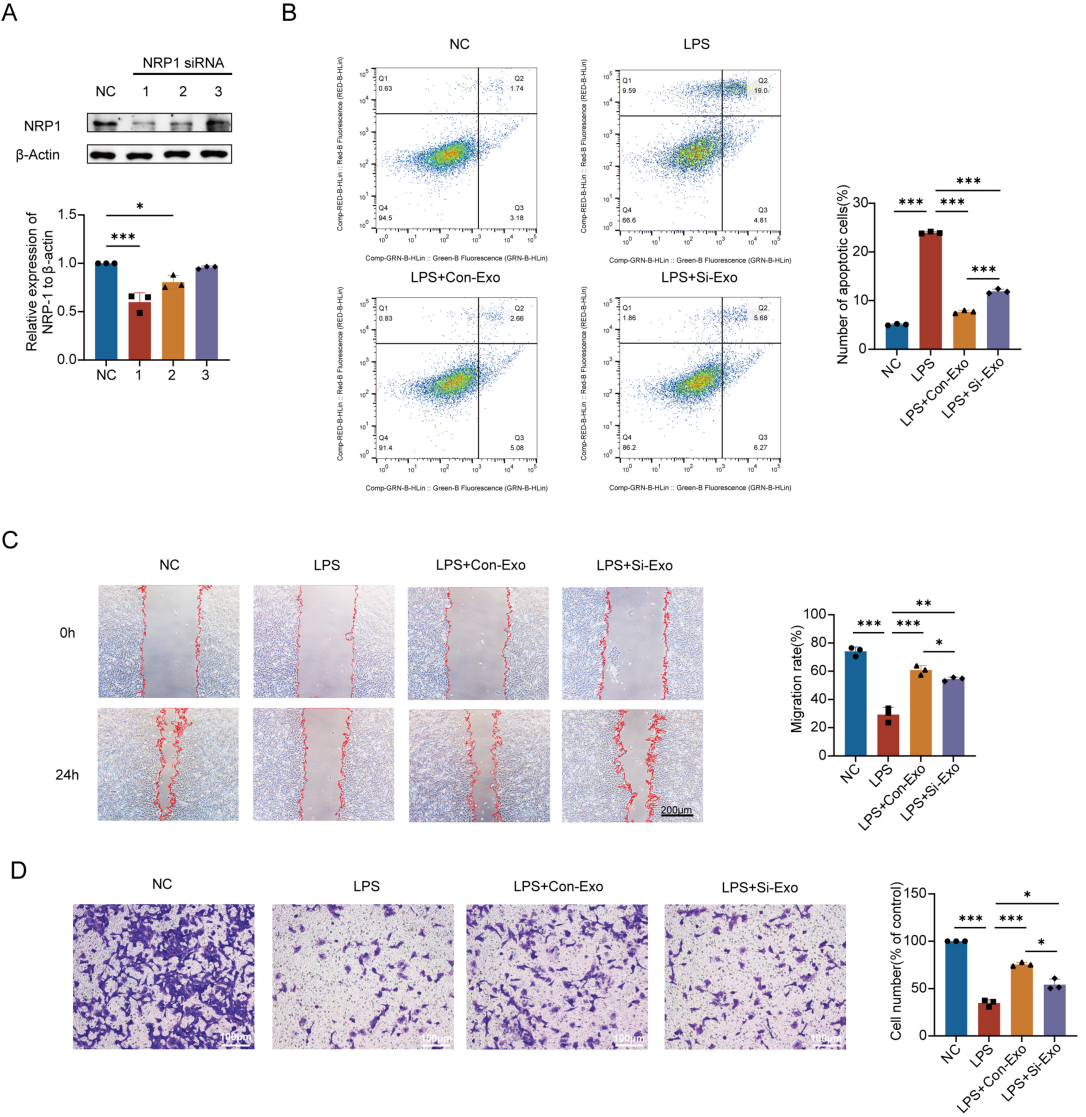
Effect of down-regulation of NRP1 level in MSC-Exo on vascular endothelial repair. (A) Western blot screening identified siRNA1 as the most effective knockdown sequence among three candidate siRNA construct (siRNA1-siRNA3); (B) FCM showed that downregulation of NRP1 in MSC-Exo attenuated its anti-apoptotic effect; (C) Wound healing assay shows that NRP1 knockdown in MSC-Exo reduces its ability to promote migration of injured PMVECs; (D) Transwell migration assay confirmed that NRP1 knockdown in MSC-Exo diminished its pro-migratory effect. Scale bar = 50 μm. All experiments were independently repeated three times. Data are presented as mean ± SD. Statistical analysis was performed using one-way ANOVA followed by Tukey’s multiple comparisons test. ^*^P < 0.05; ^**^P < 0.01; ^***^P < 0.001. NRP1: Neuropilin-1; MSCs: mesenchymal stem cells; MSC-Exo: mesenchymal stem cell-derived exosomes; siRNA: small interfering RNA; Si-Exo: exosomes derived from NRP1-silenced mesenchymal stem cells; Con-Exo: exosomes derived from control mesenchymal stem cells; LPS: lipopolysaccharide; PMVECs: pulmonary microvascular endothelial cells; FCM: flow cytometry; NC: normal control; SD: standard deviation; ANOVA: analysis of variance.

Western blot screening identified NRP1 siRNA #1 (rNRP1-si1) as the most effective knockdown sequence, with a knockdown efficiency of about 40%, significantly reducing NRP1 protein levels in MSCs [Figure 4A]. Western blot analysis further confirmed that NRP1 expression was significantly reduced in exosomes derived from siNRP1-transfected MSCs compared with non-targeting siRNA controls [Supplementary Figure 3C], indicating successful knockdown of NRP1 in MSC-Exo. Flow cytometry analysis showed that treatment with siNRP1-transfected MSC-Exo reduced PMVEC apoptosis, by approximately 12.1% compared to the LPS group, although the apoptosis rate remained higher than that of the non-targeting siRNA group [Figure 4B].

Similarly, the colony formation assay revealed more densely distributed purple colonies in the siNRP1 group, indicating a significant increase in colony area compared to the LPS group, but the area still remained lower than that of the non-targeting control [Supplementary Figure 3D]. These findings suggest that NRP1 partially regulates the anti-apoptotic and pro-proliferative effects of MSC-Exo on injured PMVECs, consistent with previous results.

Scratch wound assay showed that treatment with siNRP1-transfected MSC-Exo for 24 h improved wound closure, with PMVEC migration area increased by approximately 25.2% compared to the LPS group, though still about 6.3% lower than that of the non-targeting siRNA group [Figure 4C]. Consistently, Transwell migration assay revealed a significant increase in cell migration compared to the LPS group, but the migration rate remained lower than that of the non-targeting control [Figure 4D]. These results indicate that NRP1 partially regulates the pro-migratory effects of MSC-Exo on injured PMVECs, aligning with previous findings.

Collectively, these data suggest that NRP1 contributes to MSC-Exo-mediated regulation of PMVEC apoptosis, proliferation, and migration, providing a foundation for further investigation.

### NRP1 affects apoptosis, migration proteins and vascular permeability through the p130Cas/MMPs pathway

**Figure 5:**
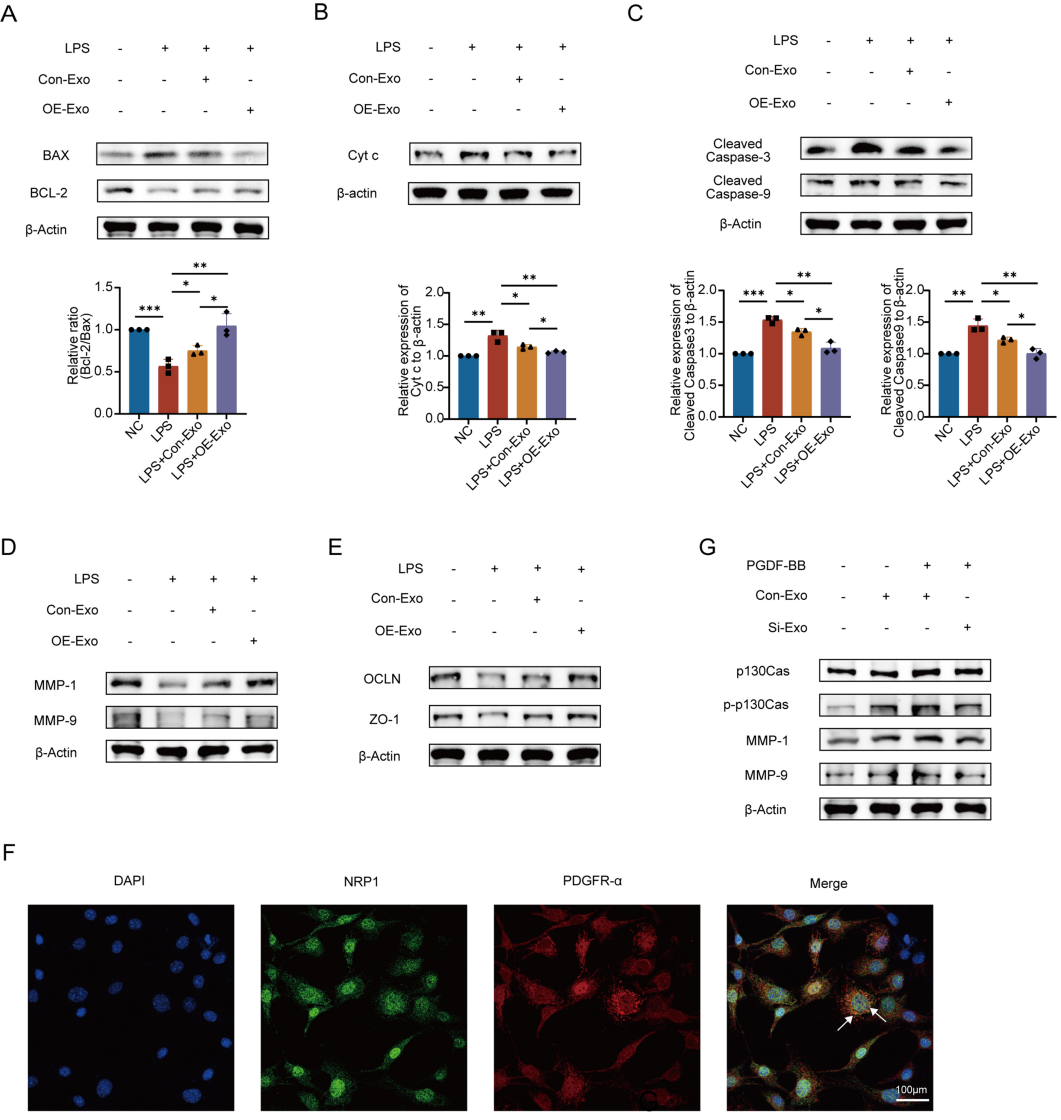
NRP1 affects apoptosis, migration proteins, and vascular permeability through the p130Cas/MMPs pathway. (A) Transfection with NRP1-overexpressing plasmid rescued LPS-induced imbalance in the Bcl-2/Bax ratio. Quantitative analysis of the Bcl-2/Bax ratio; (B) Reduced Cyt c expression following upregulation of the Bcl-2/Bax ratio. Quantitative analysis of Cyt c band intensity; (C) Downregulated levels of Cleaved Caspase-3/9. Quantitative analysis of Cleaved Caspase-3 band intensity. Quantitative analysis of Cleaved Caspase-9 band intensity. All experiments were independently repeated three times; (D) NRP1-overexpressing MSC-Exo significantly promoted MMP1 and MMP9 expression; (E) Tight junction-associated proteins OCLN and ZO-1 were synchronously upregulated; (F) Immunofluorescence confirmed co-localization of NRP1 with PDGFR-α; (G) Western blot validated NRP1-mediated activation of the p130Cas/MMPs signaling axis in combination with exogenous PDGF-BB (30 ng/mL). All experiments were independently repeated three times. Data were presented as mean ± SD. Statistical analysis was performed using one-way ANOVA followed by Tukey’s multiple comparisons test. ^*^P < 0.05; ^**^P < 0.01; ^***^P < 0.001. NRP1: Neuropilin-1; MSC-Exo: mesenchymal stem cell-derived exosomes; Con-Exo: exosomes derived from control mesenchymal stem cells; OE-Exo: exosomes derived from NRP1-overexpressing mesenchymal stem cells; Si-Exo: exosomes derived from NRP1-silenced mesenchymal stem cells; LPS: lipopolysaccharide; NC: normal control; Bcl-2: B-cell lymphoma 2; Bax: Bcl-2-associated X protein; Cyt c: cytochrome c; MMP-1: matrix metalloproteinase-1; MMP-9: matrix metalloproteinase-9; OCLN: occludin; ZO-1: zonula occludens-1; PDGFR-α: platelet-derived growth factor receptor alpha; PDGF-BB: platelet-derived growth factor-BB; p130Cas: Crk-associated substrate (130 kDa); p-p130Cas: phosphorylated Crk-associated substrate; DAPI: 4’,6-diamidino-2-phenylindole; SD: standard deviation; ANOVA: analysis of variance.

Building on prior evidence that NRP1 levels in MSC-Exo regulate PMVEC migration, proliferation, and apoptosis post-LPS injury, we investigated NRP1’s modulation of canonical apoptotic signaling via Western blotting. NRP1-overexpressing MSC-Exo significantly downregulated pro-apoptotic Bax while upregulating anti-apoptotic Bcl-2 in PMVECs [Figure 5A]. This rebalancing of the Bcl-2/Bax ratio attenuated mitochondrial Cyt c release [Figure 5B], thereby substantially suppressing cleavage/activation of Caspase-9 and Caspase-3 [Figure 5C]. These results demonstrate that NRP1 mediates MSC-Exo’s anti-apoptotic effects by orchestrating the Bcl-2/Bax-Caspase cascade in LPS-injured endothelium.

As core members of the metalloproteinase family, matrix metalloproteinases (MMPs) orchestrate tissue remodeling and cell migration by degrading extracellular matrix (ECM) components like collagen and laminin. Building on this, we investigated NRP1’s regulation of MMPs and TJ proteins. Western blot analysis demonstrated that NRP1-overexpressing MSC-Exo significantly upregulated migration-associated MMP1/9 while enhancing expression of TJproteins ZO-1 and OCLN in PMVECs [Figure 5D and E]. These findings suggest that NRP1 plays an important role in regulating endothelial barrier integrity, simultaneously facilitating cell migration through MMP1/9 induction and reinforcing intercellular junctions via ZO-1/OCLN upregulation.

Immunofluorescence co-staining showed partial spatial overlap of NRP1 and PDGFR-α at the cell membrane in PMVECs [Figure 5F], indicating a potential interaction consistent with previous reports. Previous studies have reported that NRP1 can regulate PDGF signaling and receptor activity in vascular cells, supporting a functional link between these pathways^[18,19]^. Subsequent immunoblotting demonstrated that NRP1 overexpression synergizes with PDGF-BB (30 ng/mL) to markedly enhance phosphorylation of p130Cas (p-p130Cas), with concomitant upregulation of phospho-ERK and MMP1/9. While PDGF-BB alone moderately activated p130Cas, combined NRP1/PDGF-BB treatment produced significantly amplified signaling [Figure 5G]. These findings indicate that MSC-Exo-delivered NRP1 complexes with PDGFR-α on PMVECs, enhancing PDGF-BB signaling to activate the p130Cas/MMP axis and drive endothelial migration repair.

### NRP1 can significantly alleviate pulmonary vascular hyperpermeability induced by LPS in ALI rats

**Figure 6:**
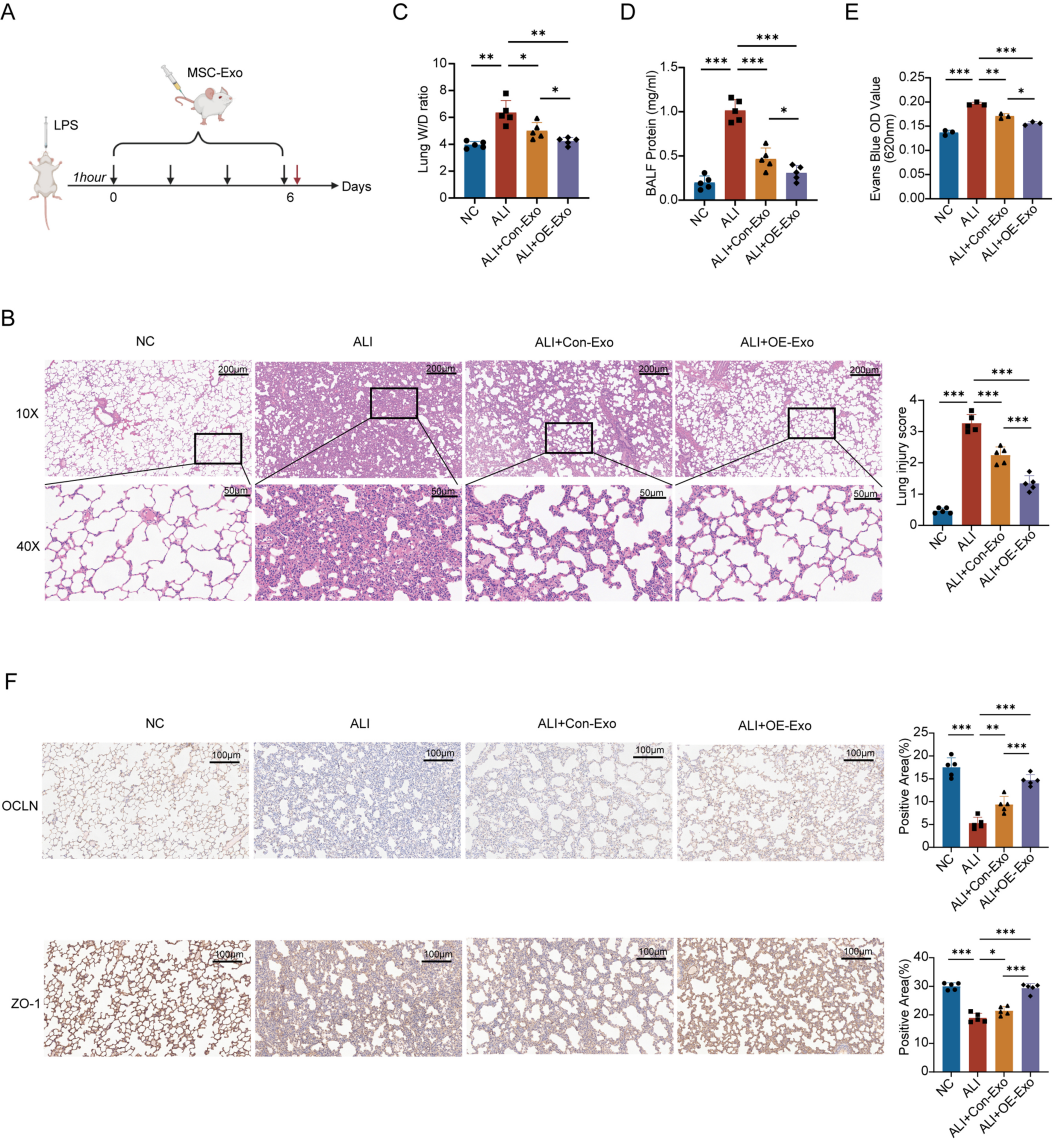
NRP1 can significantly alleviates pulmonary vascular hyperpermeability induced by LPS in ALI rats. (A) Establishment of rat ALI model and validation of MSC-Exo *in vivo*; (B) Histopathological changes and injury severity in lung tissues under different treatments were evaluated by H&E stained sections. Images reveal severe LPS-induced lung injury, while the therapeutic efficacy of OE-Exo surpasses that of conventional MSC-Exo. Quantitative analysis of ung tissue injury scores (*n* = 5). All experimental data were independently repeated three times; (C) The W/D weight ratio demonstrates the severity of pulmonary edema (*n* = 5); (D) Total protein concentration in BALF reflects lung vascular barrier integrity (*n* = 5); (E) Evans blue dye assay analyzes lung vascular barrier function via measurement of OD values (*n* = 3). All experimental data were independently repeated three times; (F) Immunohistochemical staining of OCLN and ZO-1 in lung tissue sections. Quantitative analysis of OCLN-positive immunoreactivity and ZO-1-positive immunoreactivity. All experimental data were independently repeated three times. Data are presented as mean ± SD. Statistical analysis was performed using one-way ANOVA followed by Tukey’s multiple comparisons test. ^*^P < 0.05; ^**^P < 0.01; ^***^P < 0.001. NRP1: Neuropilin-1; LPS: lipopolysaccharide; ALI: acute lung injury; MSC-Exo: mesenchymal stem cell-derived exosomes; Con-Exo: exosomes derived from control mesenchymal stem cells; OE-Exo: exosomes derived from NRP1-overexpressing mesenchymal stem cells; H&E: hematoxylin and eosin; W/D: wet-to-dry; BALF: bronchoalveolar lavage fluid; OD: optical density; OCLN: occludin; ZO-1: zonula occludens-1; NC: normal control; SD: standard deviation; ANOVA: analysis of variance.

To evaluate the therapeutic effects of MSC-Exo *in vivo*, we established an ALI model in rats via intratracheal instillation of LPS. Control animals received an equal volume of LPS-free PBS. At 1-h post-modeling, rats were administered 200 μg of MSC-Exo or vehicle control via tail vein injection. This treatment was repeated every 48 h for a total of four administrations, continuing through day 6 post-modeling. On day 6, five randomly selected rats per group were euthanized by CO_2_ asphyxiation. Lung tissues were harvested for histopathological and permeability analyses, while BALF was collected for protein quantification [Figure 6A].

H&E staining demonstrated that NRP1-overexpressing exosomes (OE-Exo) substantially reversed LPS-induced ALI pathology - including alveolar disruption, neutrophil infiltration, and hemorrhage - achieving near-normal histology. Control exosomes (Control-Exo) showed partial efficacy but significantly less structural preservation than OE-Exo (*P* < 0.01). Blinded semi-quantitative analysis confirmed OE-Exo’s superiority (68% greater improvement *vs*. Control-Exo, *P* < 0.01), establishing NRP1 upregulation as critical for enhancing exosome-mediated alveolar repair in ALI [Figure 6B].

Disruption of the alveolar-capillary barrier elevated pulmonary vascular permeability in ALI, evidenced by increased lung W/D ratios and BALF protein (*P* < 0.01 *vs*. controls; Figure 6C and D). While both exosome treatments attenuated these markers, NRP1-OE-Exo showed a more pronounced protective effect, resulting in significantly lower W/D ratios (*P* < 0.05) and BALF protein levels (*P* < 0.01). Complementary EB assays further supported this trend, with OE-Exo reducing dye extravasation by 36.2% compared with controls (*P* < 0.01; Figure 6E), indicating improved vascular barrier function. These results suggest that NRP1 contributes to the enhanced endothelial protective effects of MSC-Exo.

TJ critically maintain vascular barrier integrity by regulating paracellular pathways. Immunohistochemical analysis of lung sections revealed dense brown staining for TJ proteins OCLN and ZO-1 in normal controls, with ZO-1 signal intensity exceeding that of OCLN. LPS-induced ALI severely disrupted TJ integrity, characterized by markedly reduced protein expression (< 22% positive area *vs*. controls, *P* < 0.01) and extensive blue-negative regions. While both exosome treatments upregulated TJ proteins, OE-Exo (NRP1-overexpressing) significantly outperformed Control-Exo, restoring near-normal OCLN/ZO-1 expression (1.8-fold increased *vs*. Control-Exo, *P* < 0.01) with broader positive staining distribution [Figure 6F]. This demonstrates that NRP1 in MSC-Exo effectively repairs endothelial barrier damage by enhancing TJ protein expression. Collectively, our *in vivo* data establish NRP1 as the pivotal effector protein in MSC-Exo that ameliorates LPS-induced pulmonary vascular hyperpermeability.

## DISCUSSION

ALI/ARDS are characterized by alveolar-capillary barrier disruption with vascular leakage and pulmonary edema; however, effective pharmacological therapies remain lacking, highlighting the urgent need for novel strategies targeting endothelial injury and repair. MSC-Exo have emerged as a promising cell-free therapeutic platform due to their capacity to modulate immune responses, promote tissue repair, and restore vascular barrier integrity. Emerging evidence further demonstrates that MSC-Exo improve endothelial function by regulating intercellular junctions, mitochondrial homeostasis, and cytoskeletal dynamics, thereby mitigating vascular leakage^[20]^.

Accumulating evidence supports a critical role of NRP1 in maintaining vascular homeostasis. Bosseboeuf *et al*. demonstrated that NRP1 interacts directly with vascular endothelial cadherin (VE-cadherin) and transforming growth factor beta receptor 2 (TGFBR2), stabilizing endothelial junctions and suppressing inflammation under hemodynamic stress - a mechanism highly relevant to ALI pathophysiology^[21]^. In line with this, our study shows that upregulation of NRP1 in MSC-Exo significantly suppressed LPS-induced endothelial apoptosis, enhanced proliferative capacity, and restored migration via activation of the p130Cas/MMP signaling axis and cytoskeletal remodeling. In addition, emerging evidence suggests that NRP1 coordinates VEGF- and PDGF-mediated signaling pathways to regulate endothelial survival, permeability, and angiogenic responses under inflammatory conditions, further supporting its central role in vascular repair.

Although our findings identify NRP1 as a key mediator, exosomal cargo is inherently complex and multifunctional. The observed protective effects are unlikely to be solely attributed to NRP1, as other proteins and noncoding RNAs may exert complementary or synergistic roles in endothelial regulation. Therefore, NRP1 should be considered a critical contributor rather than the sole regulator, and further studies are required to define its relative contribution within the broader exosomal network.

Recent proteomic analyses suggest that protein cargoes [e.g., angiopoietin-like 4 (ANGPTL4), Ras-related C3 botulinum toxin substrate 1 (Rac1)] may directly modulate vascular integrity, yet functional validation remains limited. Our study bridges this critical knowledge gap through integrated proteomic and functional analyses of MSC-Exo. While most existing studies focus on noncoding RNAs, emerging evidence highlights that exosomal proteins - such as ANGPTL4, HSP70, and cytoskeleton-associated regulators - play indispensable roles in endothelial barrier regulation^[22]^. Here, we identify NRP1 as a key protein cargo that coordinates apoptotic balance via Bcl-2/Bax modulation and promotes endothelial migration through the p130Cas/MMPs signaling pathway. Validation in LPS-induced ALI models further confirmed that NRP1-enriched exosomes significantly restore vascular barrier integrity, thereby supporting a protein-centric therapeutic strategy that complements RNA-based mechanisms.

Functionally, NRP1-OE-Exo outperformed Control-Exo in reducing vascular leakage, restoring TJ protein expression (ZO-1 and OCLN), and alleviating histopathological damage in LPS-induced ALI rat models. These findings are consistent with those of Liu *et al*., who showed that MSC-Exo mitigate alveolar macrophage pyroptosis and promote resolution of inflammation in ALI through mitochondrial transfer^[23]^. Notably, the ability of NRP1 to bind PDGFR-α and enhance PDGF-BB signaling amplifies downstream activation of p130Cas, a scaffold protein critical for cell adhesion and directional migration, thereby promoting endothelial regeneration^[9]^.

Furthermore, liraglutide preconditioning enriched NRP1 levels in exosomes and enhanced their therapeutic efficacy. This finding is consistent with Yang *et al*., who reported that liraglutide protects MSCs from LPS-induced apoptosis via PKA/β-catenin signaling, thereby optimizing their secretome for vascular repair^[8]^. These results suggest that pharmacological preconditioning may represent an effective strategy to improve exosome quality and functional consistency.

The modulation of endothelial permeability is central to ALI therapy. A recent randomized controlled trial demonstrated that MSC-derived extracellular vesicles significantly reduced ARDS severity by improving alveolar barrier function^[24]^. Recent clinical studies of MSC-derived extracellular vesicles have suggested favorable safety profiles and potential improvements in oxygenation and inflammatory responses in early-phase trials^[25]^. Similarly, our data provide compelling evidence that protein cargo - specifically NRP1 - can be harnessed to potentiate exosome-based therapy. This protein-centric strategy complements existing RNA-based approaches, such as hsa-miR-148a-3p targeting ROCK1 to reinforce endothelial junctions^[5]^, and broadens the mechanistic landscape of EV-based interventions. Endothelial cell migration is context-dependent. In ALI, increased PMVEC migration is generally regarded a reparative process that contributes to endothelial regeneration rather than barrier disruption. Therefore, the enhanced migration observed in this study reflects improved endothelial repair capacity.

Moreover, by integrating proteomic screening and functional validation, our study supports a broader shift toward exosome engineering to enhance therapeutic precision and reproducibility. The upregulation of NRP1 not only may reduce batch-to-batch variability but also provides mechanistic clarity regarding endothelial repair pathways, addressing a major limitation in current MSC-based applications.

Despite these promising findings, several limitations should be acknowledged. Exosome-based therapies are subject to batch-to-batch variability and challenges in large-scale production and standardization. In addition, exosome preparations may contain co-isolated contaminants, such as protein aggregates or lipoproteins, which could influence functional outcomes. The heterogeneity of exosomal cargo may also affect biological activity and reproducibility. Furthermore, the safety profile, potential off-target effects, and long-term efficacy of exosome administration remain to be fully elucidated, particularly in the context of systemic delivery.

Alternative interpretations should also be considered, as the observed effects may involve indirect mechanisms, including modulation of inflammatory responses or ECM dynamics, rather than solely direct NRP1 signaling. From a translational perspective, challenges such as large-scale production, standardized purification, and optimization of delivery strategies remain to be addressed. Therefore, further studies are required to validate the efficacy, safety, and scalability of this approach prior to clinical application.

In conclusion, our findings underscore the therapeutic value of exosome-delivered NRP1 in restoring endothelial barrier integrity during ALI. Mechanistically, NRP1 suppresses apoptosis via modulation of the Bcl-2/Bax-caspase cascade and promotes migration via PDGF-BB/p130Cas/MMP signaling. *In vivo*, NRP1-enriched exosomes significantly reduce pulmonary hyperpermeability and lung injury severity. These results support the development of NRP1-targeted, protein-enriched exosome therapies as a viable strategy for vascular repair in inflammatory lung diseases.

## DECLARATIONS

### Availability of data and materials

The data underlying this article are available in the article and in its online Supplementary Materials.

### AI and AI-assisted tools statement

During the preparation of this manuscript, the AI tool ChatGPT (GPT-5.5, OpenAI) was used solely for language editing. The tool did not influence the study design, data collection, analysis, interpretation, or the scientific content of the work. All authors take full responsibility for the accuracy, integrity, and final content of the manuscript.

### Copyright

© The Author(s) 2026.

## References

[1] (2021). Bossardi Ramos R, Adam AP. Molecular mechanisms of vascular damage during lung injury. Adv Exp Med Biol.

[2] Long ME, Mallampalli RK, Horowitz JC (2022). Pathogenesis of pneumonia and acute lung injury. Clin Sci.

[3] Mowery NT, Terzian WTH, Nelson AC (2020). Acute lung injury. Curr Probl Surg.

[4] (2025). Renard Triché L, Jabaudon M, Chevret S, Constantin JM, Pereira B, Molinari N. Post-hoc mediation analysis of two biomarkers, and survival in acute respiratory distress syndrome. Sci Rep.

[5] Lv Y, Yu W, Xuan R, Yang Y, Xue X, Ma X (2024). Human placental mesenchymal stem cells-exosomes alleviate endothelial barrier dysfunction via cytoskeletal remodeling through hsa-miR-148a-3p/ROCK1 pathway. Stem Cells Int.

[6] Averyanov A, Koroleva I, Konoplyannikov M (2020). First-in-human high-cumulative-dose stem cell therapy in idiopathic pulmonary fibrosis with rapid lung function decline. Stem Cells Transl Med.

[7] Hoang DM, Pham PT, Bach TQ (2022). Stem cell-based therapy for human diseases. Signal Transduct Target Ther.

[8] Yang X, Ma X, Don O (2020). Mesenchymal stem cells combined with liraglutide relieve acute lung injury through apoptotic signaling restrained by PKA/β-catenin. Stem Cell Res Ther.

[9] Wu Q, Fu S, Xiao H (2023). Advances in extracellular vesicle nanotechnology for precision theranostics. Adv Sci.

[11] Lotfy A, AboQuella NM, Wang H (2023). Mesenchymal stromal/stem cell (MSC)-derived exosomes in clinical trials. Stem Cell Res Ther.

[12] Zhao R, Wang L, Wang T, Xian P, Wang H, Long Q (2022). Inhalation of MSC-EVs is a noninvasive strategy for ameliorating acute lung injury. J Control Release.

[13] Zhou Z, Wu S, Li Y, Shao P, Jiang J (2025). Inhibition of macrophage polarization and pyroptosis in collagen-induced arthritis through MSC-exo and ginsenoside Rh2. Arthritis Res Ther.

[14] Kundu D, Shin SY, Chilian WM, Dong F (2024). The potential of mesenchymal stem cell-derived exosomes in cardiac repair. Int J Mol Sci.

[15] Wang C, Zhou H, Wu R (2023). Mesenchymal stem cell-derived exosomes and non-coding RNAs: regulatory and therapeutic role in liver diseases. Biomed Pharmacother.

[16] (2022). Yousefi Dehbidi M, Goodarzi N, Azhdari MH, Doroudian M. Mesenchymal stem cells and their derived exosomes to combat Covid-19. Rev Med Virol.

[17] Valiukevičius P, Mačiulaitis J, Pangonytė D (2023). Human placental mesenchymal stem cells and derived extracellular vesicles ameliorate lung injury in acute respiratory distress syndrome murine model. Cells.

[18] Pellet-Many C, Frankel P, Evans IM, Herzog B, Jünemann-Ramírez M, Zachary IC (2011). Neuropilin-1 mediates PDGF stimulation of vascular smooth muscle cell migration and signalling via p130Cas. Biochem J.

[19] Muhl L, Folestad EB, Gladh H (2017). Neuropilin 1 binds PDGF-D and is a co-receptor in PDGF-D-PDGFRβ signaling. J Cell Sci.

[20] Worthington EN, Hagood JS (2020). Therapeutic use of extracellular vesicles for acute and chronic lung disease. Int J Mol Sci.

[21] Bosseboeuf E, Chikh A, Chaker AB (2023). Neuropilin-1 interacts with VE-cadherin and TGFBR2 to stabilize adherens junctions and prevent activation of endothelium under flow. Sci Signal.

[22] Zhou L, Luo H, Lee JW (2022). Role of extracellular vesicles in lung diseases. Chin Med J.

[23] Liu P, Yang S, Shao X (2024). Mesenchymal stem cells-derived exosomes alleviate acute lung injury by inhibiting alveolar macrophage pyroptosis. Stem Cells Transl Med.

[24] Zarrabi M, Shahrbaf MA, Nouri M (2023). Allogenic mesenchymal stromal cells and their extracellular vesicles in COVID-19 induced ARDS: a randomized controlled trial. Stem Cell Res Ther.

[25] Wang F, Li Y, Wang B, Li J, Peng Z (2023). The safety and efficacy of mesenchymal stromal cells in ARDS: a meta-analysis of randomized controlled trials. Crit Care.

[26] Liang D, Liu C, Yang M (2023). Mesenchymal stem cells and their derived exosomes for ALI/ARDS: a promising therapy. Heliyon.

