# Supplementary material for: Neuropilin-1 enriched mesenchymal stem cell derived-exosomes alleviate vascular hyperpermeability in acute lung injury
Source: Extracell Vesicles Circ Nucl Acids. 2026 Jun 16;7(2):904–26. doi: 10.20517/evcna.2025.169 (PMC13367150; doi:10.20517/evcna.2025.169)
Supplement: Supplementary file 1 [file evcna-7-2-904-SupplementaryMaterials.zip › evcna60169-SupplementaryMaterials/Supplementary Figures 1-3.pdf]

Supplementary Materials

Neuropilin-1 enriched mesenchymal stem cell derived-exosomes alleviate vascular hyperpermeability in acute lung injury

Qinyi Deng[1,2,#], Yifan Lu[1,#], Linyan Yuan[2], Shiyu Gao[2], Xiaoyan Hu[1], Van Minh Le[3], Xiaoyan Chen[4], Xin Liang[2], Yun Feng[1]

[1]Department of Respiratory and Critical Care Medicine, Ruijin Hospital, School of Medicine, Shanghai Jiao Tong University, Shanghai 200025, China.
[2]State Key Laboratory of Bioreactor Engineering & Shanghai Key Laboratory of New Drug Design, School of Pharmacy, East China University of Science and Technology, Shanghai 200237, China.
[3]Research Center for Discovery and Development of Healthcare Products, Vietnam National University Ho Chi Minh City, Ho Chi Minh City 700000, Vietnam.
[4]Department of Pathology, Ruijin Hospital, School of Medicine, Shanghai Jiao Tong University, Shanghai 20025, China.
[#]These authors contributed equally to this work.

Correspondence to: Dr. Yun Feng, Department of Respiratory and Critical Care Medicine, Ruijin Hospital, School of Medicine, Shanghai Jiao Tong University, Shanghai 200025, China. E-mail: fy11697@rjh.com.cn; Dr. Xin Liang, State Key Laboratory of Bioreactor Engineering & Shanghai Key Laboratory of New Drug Design, School of Pharmacy, East China University of Science and Technology, Shanghai 200237, China. E-mail: xin.liang@ecust.edu.cn; Dr. Xiaoyan Chen, Department of Pathology, Ruijin Hospital, School of Medicine, Shanghai Jiao Tong University, Shanghai 20025, China. E-mail: cxy11832@rjh.com.cn

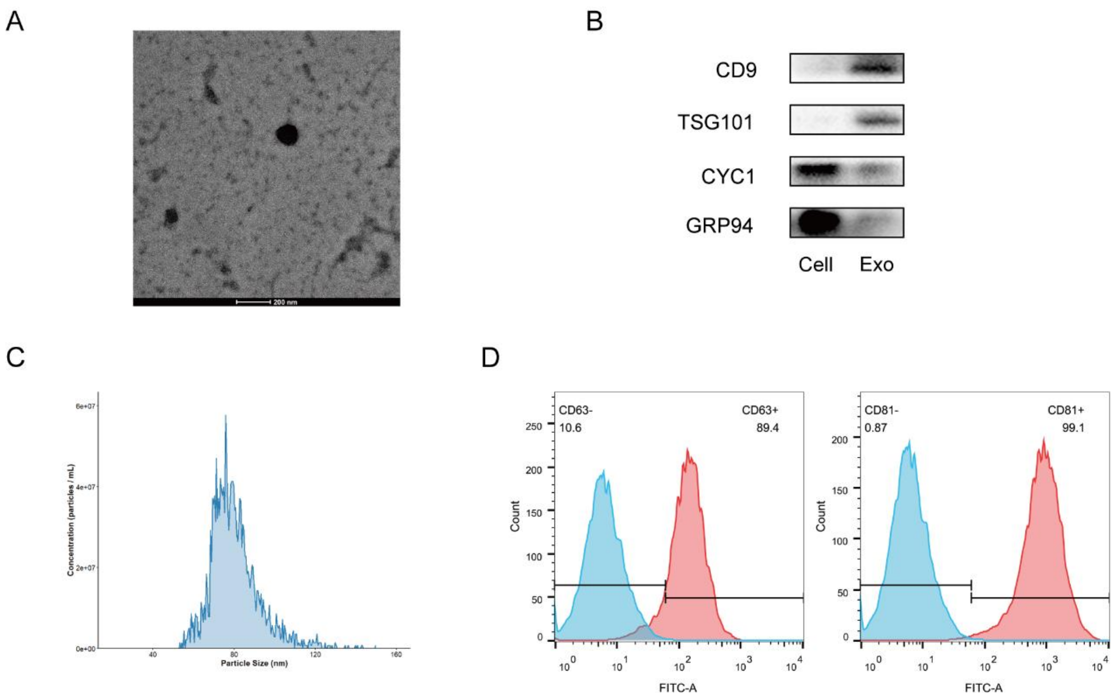

Supplementary Figure 1. (A) TEM image of MSC-Exo; (B) Western Blot analysis of surface markers and contaminant proteins in MSC-Exo; (C) Nanoparticle tracking analysis (NTA) showing particle size distribution of MSC-Exo; (D) Flow cytometry detection of MSC-Exo surface marker positivity.

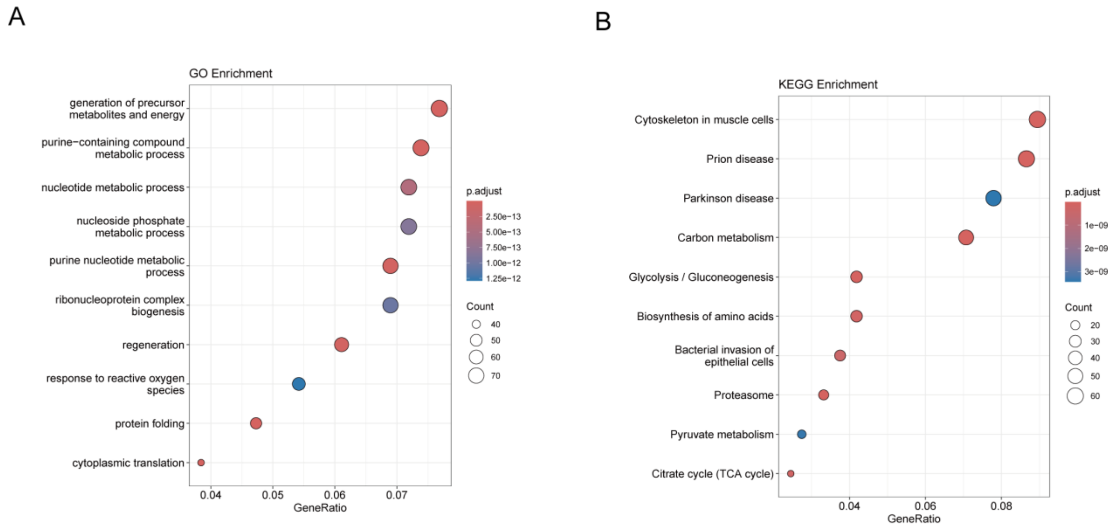

Supplementary Figure 2. (A)Bubble plot of top 10 GO-enriched terms; (B) Bubble plot of top 10 KEGG-enriched pathways. The X-axis indicates gene ratio, while the Y-axis displays functional terms (A) and pathway information (B). Bubble size corresponds to the number of enriched genes, with color intensity reflecting -log10 (*P*-value) (darker hues indicate higher significance levels). Smaller enrichment P-values denote greater statistical significance.

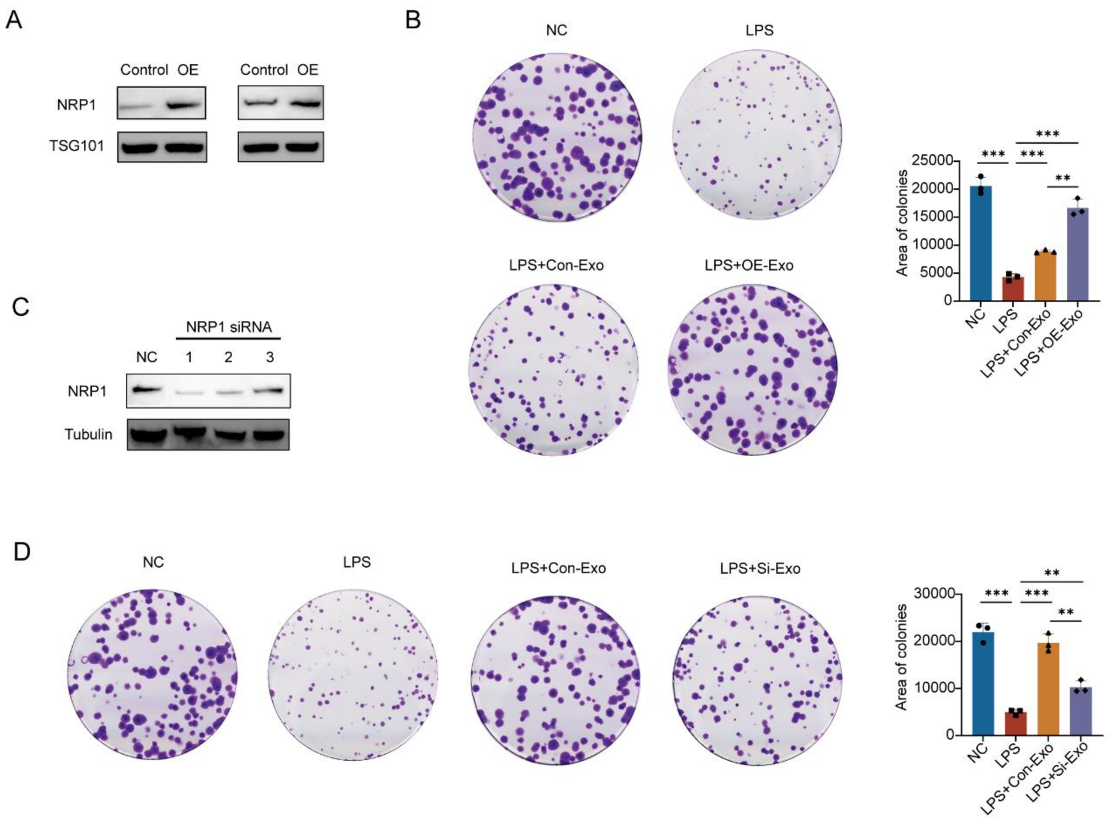

Supplementary Figure 3. (A) Western blot analysis of NRP1 expression in exosomes derived from control and NRP1-overexpressing MSCs; (B) Colony formation assay confirmed the proliferative advantage of PMVECs treated with NRP1-overexpressing MSC-Exo. All experiments were independently repeated three times; (C) Western blot screening confirmed that siRNA1 exhibited the highest knockdown efficiency among three candidate siRNA constructs. NRP1 expression was further evaluated in MSC-derived exosomes, showing a significant reduction in exosomes from siNRP1-transfected MSCs compared with non-targeting siRNA controls. All experiments were independently repeated three times; (D) Colony formation assay confirms that downregulation of NRP1 in MSC-Exo diminishes its pro-proliferative effect. All experiments were independently repeated three times.
